# Descriptive analysis of injury types and incidence during futsal preseason across different competitive levels

**DOI:** 10.3389/fspor.2024.1363006

**Published:** 2024-03-07

**Authors:** Catarina Marques, Miguel Rebelo, Rute Crisóstomo, Samuel Honório, Pedro Duarte-Mendes, João Petrica, João Serrano

**Affiliations:** ^1^ Department of Sports and Well-Being, Polytechnic Institute of Castelo Branco, Castelo Branco, Portugal; ^2^ Sport, Health & Exercise Research Unit (SHERU), Polytechnic Institute of Castelo Branco, Castelo Branco, Portugal; ^3^ Sport Physical activity and health Research & INovation cenTer (SPRINT), Castelo Branco, Portugal; ^4^ AGE.COMM- Interdisciplinary Research Unit, Polytechnic Institute of Castelo Branco, Castelo Branco, Portugal

**Keywords:** futsal, injury incidence, sports injury, level of competition, preseason

## Abstract

**Introduction:**

This study aimed to verify the typology and incidence of injury by comparing the different competitive levels of futsal during the preseason.

**Methods:**

The sample consisted of 68 senior male futsal players (24.26 ± 4.63 years). Data were collected using an injury recording grid that examined the affected body part, anatomical region, type of injury, mechanism, and severity.

**Results:**

It was found that the elite group has the lowest incidence rate of injury (4.8 injuries per 1,000 h of exposure) compared to the sub-elite (11.8 injuries per 1,000 h of exposure) and amateur groups (13.9 injuries per 1,000 h of exposure). However, at this level, there is the highest percentage of injury occurrence (38.5%), the lower limb was the most affected part of the body (30.8%), and ligament (23.1%) and muscle (15.4%) injuries are the most prevalent. The most frequent mechanism of injury was non-traumatic (30.8%), and the majority were moderate injuries in the elite (23.1%) and sub-elite (17.9%) groups and severe injuries in the amateur group (12.5%).

**Discussion:**

The amateur futsal players had the highest incidence of injury during the preseason period compared to the other competitive levels. Still, it was at the elite level where the highest percentage of injuries occurred, most of them non-traumatic and of ligament origin, primarily affecting the ankle region. The results highlight the importance of adopting specific injury prevention programs for ligament and muscle injuries during the preseason phase, regardless of the competitive level.

## 1 Introduction

Futsal is a team sport with a considerable risk of injuries, attributed to several justifiable factors, including the dynamic nature of the match, characterized by intermittent high-intensity activities and frequent physical contact, which significantly increases the risk of injury (1–5). Furthermore, the small size of the game court (40 × 20 m) increases the challenges for players, placing it among the ten highest risk team sports (6). Sports injuries have been widely studied, but in futsal, this data needs to be more comprehensive (2, 7, 8) and would be essential to help health and sports professionals implement prevention protocols (2, 9).

The preseason corresponds to the time-interval between the first training session and the first league match (10), and it is during this period that training aims to prepare and improve the players' physical condition for the competitive period (7, 11). Consequently, this phase is characterized by a greater training load compared to the rest of the season (12–14). In a broader context, the preseason aims to promote the athlete's physical adaptation, reducing the likelihood of injury and optimizing the players' participation in training sessions (11, 15). Several authors have observed a significant increase in the risk and prevalence of injury in the early stages of the season (2, 16). The higher incidence of injuries in this period can be explained by the fact that, at the start of the sporting season, training loads are considerably higher (12, 14) and, in addition, the accumulation of fatigue can contribute to a greater risk of injury during the first few weeks of competition. Furthermore, there seems to be a positive relationship between training load and the likelihood of injury, i.e., the greater the training load, the greater the possibility of injury (17).

According to the available literature, the vast majority of epidemiological studies report that futsal injuries are predominantly located in the lower limbs (18, 19), which is justified given the characteristics of the sport (20, 21). The ankle, knee and thigh are the areas of the body with the highest injury rates (2, 7, 19, 22), and ligament injuries are the most common (2, 21, 23). Regarding the mechanism of injury, non-contact injuries are the most commonly reported mechanism (7) and are generally more severe than contact injuries (24).

Despite being one of the most practiced sports worldwide, the literature points to a lack of scientific research into the injuries suffered by futsal players (2, 7, 9, 25, 26), and it is also pertinent to investigate the incidence of these injuries at different competitive levels. In this sense, the study aimed to verify the type and incidence of injuries comparing the different competitive levels of futsal and, based on their typology, to alert health and sports professionals to raise awareness and adopt preventive measures that could potentially reduce the risk of these most common injuries, particularly at this crucial stage of the season, as mentioned by other authors (7, 8).

According to the literature, we expect: (1) a greater number of injuries at the elite level of futsal compared to the lower levels, bearing in mind that the interaction between high-intensity technical gestures combined with the demands of competitive calendars and the longer exposure time to practice can make these players more susceptible to injury (27); (2) the incidence rate of injury to be higher at the amateur level due to the lack of follow-up and investment in injury prevention; and (3) a higher percentage of ligament injuries compared to any other type of injury, given their greater representation in futsal, as highlighted by several authors (8, 21, 23).

## 2 Methodology

### 2.1 Study design

This descriptive and observational study was carried out on a total of five futsal teams, one of which competes in the 1st National Division (elite group), two compete in the 2nd National Division, one competes in the 3rd National Division (sub-elite group), and one competes at District level (amateur group) in Portugal. Recordings were made from early August to the end of September 2023, during each team's pre-season period (4 weeks), respectively.

### 2.2 Participants

In this study, 68 athletes (24.26 ± 4.63 years) took part, subdivided into three groups: elite (23.77 ± 4.38 years), sub-elite (25.36 ± 4.83 years) and amateur (22.01 ± 3.55 years), and all teams had exactly the same pre-season period, that is, 4 weeks, but with different numbers of training sessions and therefore different exposure times to practice over those 4 weeks. During the preseason, the elite players had a substantially higher training load and, consequently, total exposure time than the other competitive levels (Table 1).

**Table 1:** General characterization of the participants.

Group	*N*	Age	No. of training sessions/week	Total training time/week (hours)	Total exposure time (hours)
Elite	13	23.77 ± 4.38	10	20	80
Sub-elite	39	25.36 ± 4.83	5	6.5	26
Amateur	16	22.01 ± 3.55	3	4.5	18

For the sample selection, the inclusion criteria were all senior male players duly registered with the club; and the exclusion criteria were players who were injured or in the process of recovery at the start of the research.

### 2.3 Procedures

Initially, formal and institutional contact was made with the clubs, presenting the objectives and requesting their cooperation, after which a characterization questionnaire and an informed consent form were given to the participants. The study's objectives were then explained to all the players who met the inclusion criteria, which respected and preserved all the ethical principles, norms and international standards relating to the Declaration of Helsinki and the Convention on Human Rights and Biomedicine, and was approved by the Technical-Scientific Committee of the Polytechnic Institute of Castelo Branco (20180770/CTC-IPCB/2023).

The record of injuries and exposure time was recorded daily by the physiotherapist of each club during the preseason of each club. All injuries were recorded using the injury report developed by Fuller et al. (28) and categorized according to the part of the body that suffered structural and/or functional changes, the anatomical region, the type of injury, the mechanism traumatic (results from a specific and identifiable event) or non-traumatic (results from repeated microtraumas without a single, identifiable event) and the severity [minimal (1–3 days), mild (4–7 days), moderate (8–28 days) and severe (more than 28 days)]. Total player exposure was defined as the sum of training exposure (hours).

### 2.4 Statistical analysis

The data was processed using the Statistical Package for the Social Sciences (SPSS) (v.23.0), and a descriptive analysis based on percentages was used to summarize the data collected. The injury incidence rate was calculated based on the total exposure time per 1,000 h of exposure (number of injuries × 1,000 h/exposure time) (28).

## 3 Results

Based on Table 2, we can verify that the elite group had the highest percentage of injuries (38.5%), but had the lowest incidence rate (4.8 per 1,000 h of total exposure). The lower limb was the most affected part of the body (30.8%), particularly the ankle region (23.1%). Ligament injuries predominated at an elite level, with the non-traumatic mechanism being the most notable (30.8%). The severity of the injuries was categorized as moderate, representing 23.1% of the cases.

**Table 2:** Summary of injury characteristics in futsal players.

Group	% Injury occurrence	Injury rate	Body location	Anatomic region	Type of injury	Mecanism	Severity
Elite	38.5%	4.8	Lower limb (30.8%) Trunk (7.7%)	Ankle (23.1%) Knee (7.7%) Lower back (7.7%)	Ligament (23.1%) Muscle (7.7%) Joint (7.7%)	Traumatic (7.7%) Non-Traumatic (30.8%)	Minimal (7.7%) Mild (7.7%) Moderate (23.1%)
Sub-elite	30.8%	11.8	Lower limb (25.6%) Upper limb (5.1%)	Thigh (10.3%) Groin (5.1%) Knee (2.6%) Ankle (2.6%) Foot (2.6%) Elbow (2.6%) Wrist (2.6%) Hip (2.6%)	Muscle (15.4%) Joint (7.7%) Ligament (5.1%) Bone (2.6%)	Traumatic (15.4%) Non-Traumatic (15.4%)	Mild (7.7%) Moderate (17.9%) Severe (5.1%)
Amateur	25%	13.9	Lower limb (25%)	Knee (12.5%) Thigh (6.3%) Ankle (6.3%)	Muscle (12.5%) Ligament (12.5%)	Traumatic (18.8%) Non-Traumatic (6.3%)	Mild (6.3%) Moderate (6.3%) Severe (12.5%)

As for the sub-elite group, an injury occurrence rate of 30.8% and an injury incidence rate of 11.8 per 1,000 h of total exposure were recorded, with the most significant impact on the lower limb (25.6%). The thigh was the region most affected (10.3%), and muscle injuries were the most prominent (15.4%). There was an equal distribution between traumatic and non-traumatic injury mechanisms (15.4% each). As for the severity of the injuries, similarly to the elite level, it was generally moderate, accounting for 17.9% of the cases.

Finally, at the amateur level, there was a lower percentage of injuries (25%). Still, the incidence rate was higher than the other levels (13.9 per 1,000 h of total exposure), concentrated exclusively in the lower limb. The knee was the anatomical region most affected (12.5%), and muscle and ligament injuries were equally common (12.5% each). The severity of injuries was higher at this level, with 12.5% of cases categorized as severe.

## 4 Discussion

The study of this topic in futsal is of great importance, because it can contribute to the implementation of preventive measures that can reduce the incidence of injuries and/or mitigate their severity, particularly during the pre-season period as it is more susceptible to the appearance of injuries compared to other phases of the sporting season. With this in mind, this study aimed to analyze the sports injuries suffered by Portuguese futsal players from three different competitive levels, regarding their type and incidence, during the 2023/2024 preseason.

Regarding the competitive level, although the elite group had a higher percentage of injuries during the preseason, the injury incidence rate was the lowest when comparing the three groups (4.8 injuries per 1,000 h of exposure). The injury incidence rate in futsal, during the preseason in particular, has differed in various studies, with our results at this competitive level being closer to those obtained in the study by Lopes et al. (7), who recorded an incidence rate of 5.9 injuries per 1,000 h of exposure, and further away from those obtained by López-Segovia et al. (29), who recorded a rate of 9.9 injuries per 1,000 h of training and 61.1 injuries per 1,000 h of match. The highest injury incidence rate was found at amateur level (13.9 injuries per 1,000 h of exposure). Although there are no records in the literature of this type of group in the specific preseason period, it is essencial to note that these figures are considered high in the practice of the sport, which is not surprising given that there is insufficient preparation and injury prevention methodologies at this competitive level (9).

The fact that the elite level has a more prolonged exposure to practice (4 times longer than the other levels) also results in a higher percentage of injuries, just as Tomsovsky et al. (30) stated that greater competitiveness can result in a greater risk of injury. Furthermore, we know that there is a positive relationship between high training volume and the risk of injury, and that this excessive increase in training load is responsible for a large proportion of non-traumatic injuries (31).

Regarding the part of the body most affected, most injuries were predominantly to the lower limbs, regardless of the level of competition, which is in line with previous studies (7, 18–21, 32). Naturally, the higher percentage of injuries in this segment is justified due to the greater demand on the lower limbs in this sport (33).

Regarding anatomical regions, the majority of injuries occurred in the ankle (23.1%), knee (12.5%), and thigh (10.3%). This observation aligns with the findings of Lopes et al. (7) and is consistent with trends highlighted by other authors (2, 8, 18–20, 22, 26). Nevertheless, the high percentage of injuries, especially to the ankle and knee, may be due to the particularities of futsal (34), which place a high level of stress on these structures by requiring constant acceleration and deceleration, tackling, jumping and changes of direction (35–37). The most common type of injury in this study was ligament damage in the elite group, followed by muscle damage in the sub-elite group, as seen previously (2, 7, 21–23).

Regarding the mechanism of injury, non-traumatic injuries were identified as the most common in futsal, consistent with previous findings (7, 29, 30, 38). Concerning the severity of the injuries, we found that the majority were moderate, followed by severe, as other authors have found (8, 39, 40), contrarily to other studies, which indicate that the most futsal injuries are minor (18–20). According to Lopes et al. (7), this data results from the competitive demands of Portuguese futsal, which require players to work very close to their maximum performance. Even so, only at amateur level was there a higher percentage of severe injuries, which may indicate a need for more management and investment in the medical department of amateur clubs.

Finally, the main objective of the preseason in futsal is to prepare players mentally and physically for the competitive season. However, these four weeks of preparation can be extremely demanding from a physical point of view due to the high training loads, resulting in a high percentage of injuries (12, 14, 17) and naturally putting all the planning and investment for the rest of the sporting season at risk. This underscores a crucial caution for professionals tasked with managing training loads during this stage. Our findings revealed that 30.9% of players experienced injuries in this period.

The major limitations of the study were the small sample size at the various competitive levels, which means we can't draw general conclusions for the whole sport, and the fact that injuries were recorded by departments independent from the research, which does not allow us to exclude the possibility that not all injuries were reported, especially minimal and mild injuries. Despite these limitations, this study has provided important information on futsal injuries, particularly at different levels of competition, contributing to the development of the sport. It is therefore suggested that it be replicated with other samples, in more significant numbers and with a correlation to the players' initial physical condition and its impact on the risk of developing an injury.

## 5 Conclusion

The amateur futsal players had the highest incidence of injury during the preseason period compared to the other competitive levels. Still, it was at the elite level where the highest percentage of injuries occurred, most of them non-traumatic and of ligament origin, primarily affecting the ankle region.

The results highlight the importance of adopting specific injury prevention programs for ligament and muscle injuries during the preseason phase, regardless of the competitive level. Finally, differentiating the type and incidence of injury by level of competition can help physical trainers and physiotherapists recognize injuries at each competitive level and devise prevention strategies to optimize the futsal player's sporting performance, essentially at amateur level.

## Data Availability

The raw data supporting the conclusions of this article will be made available by the authors, without undue reservation.
